# The Impact of a Neurocollaborative Theraplay^®^-Informed Intervention on the Presentations of Developmental Trauma and Attachment Difficulties in a Sub-Group of Autistic Looked after Children: a Retrospective Service Evaluation

**DOI:** 10.1007/s40653-026-00964-6

**Published:** 2026-09-02

**Authors:** Jack Purrington, Gemma Hartley, Alan Price, Elspeth Bass, Chloe Godfrey, Jacqueline Lynch, Raja Mukherjee

**Affiliations:** 1 Chrysalis Associates, York, UK; 2 York CAMHS Neurodevelopmental Assessment Team, York, UK; 3 School of Health and Society, University of Salford, Salford, UK; 4 Surrey and Borders Partnership NHS Foundation Trust, Leatherhead, UK

**Keywords:** Autism, Trauma, Attachment Disruption, Theraplay

## Abstract

The intersection between autism, developmental trauma, and attachment disruption contributes to unique difficulties for care experienced autistic children. However, understanding regarding the efficacy of Theraplay^®^ for this cohort is lacking. This study evaluated the impact of a 30-hour neuro-collaborative Theraplay^®^-informed psychotherapeutic intervention on the presentations of developmental trauma and attachment difficulties in care experienced autistic children compared to a non-autistic group. Between April 2020 and April 2024, intervention efficacy was evaluated utilising a mixed design with an autistic group (*N* = 35) and non-autistic group (*N* = 172). The 30-hour intervention included 10-hours of therapy informed by Theraplay^®^ principles for parents and children, 10-hours of sensory regulation delivered directly to children or psycho-educationally to parents, and 10-hours of therapeutic parenting informed by the principles of dyadic developmental parenting. Therapeutic outcomes were measured at pre- and post-therapy utilising the Trauma Symptom Checklist for Young Children (TSCYC), the Child Behaviour Checklist (CBCL), the Behaviour Rating Inventory of Executive Function 2nd Edition (BRIEF-2), and the Assessment Checklist for Children (ACC). Paired t-tests showed that post-treatment scores significantly improved compared to pre-treatment scores in the full cohort and non-autistic group in the TSCYC and CBCL and analyses of covariance (ANCOVA) controlling for age and gender showed no difference in treatment outcome between autistic and non-autistic children. While t-tests did not demonstrate significant improvements in the smaller autistic group, there was movement in the direction of improvement accompanied by encouraging effect sizes. The 30-hour neuro-collaborative Theraplay^®^-informed intervention was shown to be effective for children with a history of developmental trauma and attachment disruption, and it may be effective for children who are also autistic but further studies with larger samples will be required to confirm this. Implications for practice are discussed.

Autism spectrum disorder, henceforth referred to as ‘autism’, can be conceptualised as an organic way of being that naturally differs from societal norms and expectations (Robertson, 2009). Core characteristics of autism include a range of social communication and interaction patterns that significantly differ to non-autistic communication patterns. This is presented alongside restricted and repetitive behaviours (American Psychiatric Association, 2013).

Developmental trauma refers to experiences of physical, sexual, and emotional abuse and/or neglect perpetrated by a caregiver towards their child (Felitti et al., 1998; van der Kolk & D’Andrea, 2010). An attachment disruption can be conceptualised as a separation of a parent-child dyad that interrupts the development of a secure attachment. These experiences can result in children experiencing disorganisation and/or insecurity in their relationships with others (NICE, 2015).

It is understood that the aforementioned differences in autistic functioning can lead to a number of interpersonal difficulties (Gosling et al., 2023; Lupindo et al., 2023) that can result in or mimic attachment disruptions (Moran, 2010; Rutgers et al., 2004; Sarr et al., 2024; Zainab Al-Attar & Worthington, 2024). These difficulties can lead to autistic children being twice as likely to experience an adverse childhood experience compared to their peers (Hartley et al., 2024). Indeed, autistic children are overrepresented in the care system (Parsons et al., 2018). Autistic individuals are therefore more likely to experience traumatic early life events alongside experiencing organic interpersonal challenges with some of these traits presenting as being similar to those navigated by care experienced children who have endured developmental trauma and attachment disruptions. This is despite the two presentations having much differing foundations for autistic populations. It can subsequently be hypothesised that there may be a compounding effect between natural autistic differences and the impact of developmental trauma and attachment disruption for autistic children who have experienced these difficult early starts.

All children who have experienced developmental trauma and attachment disruptions can find it hard to develop security and trust within their social relationships (Cyr et al., 2010), often resulting in a range of social communication and interaction styles that differ from non-traumatised, securely attached peers. Equally, these children can find it increasingly challenging to regulate their behaviours (Dubowitz et al., 2002) and emotions (Milot et al., 2010) which can contribute to a range of restrictions and repetitions in behavioural patterns and presentations. The unique intersection and potentially compounding relationship between autism, developmental trauma, attachment difficulties therefore warrants additional attention.

Recent reviews of literature exploring psychotherapeutic interventions for autistic children following experiences of traumatic events detail the adapted use of trauma-focused cognitive behavioural therapy (TF-CBT; Peterson et al., 2019; Stack & Lucyshyn, 2019). Recommendations for key adaptations to the TF-CBT PRACTICE model for autistic children include: incorporating treatment goals related to autism core symptoms and associated characteristics and introducing a functional behavioural assessment, behaviour planning and behavioural skill training, parent and child support between sessions, and programming for generalisation and maintenance. Seperately, Ostfeld-Etzion (2024) presented a case-report which examined the use of play therapy for an autistic fourth-grade child (aged 9–10 years) with post-traumatic stress disorder (PTSD) presentations. Similarly, this report recommends adaptations to traditional approaches within play therapy and presents promising findings. It is worth noting that these intervention findings are not specific to experiences of developmental trauma as previously defined within this study. One study which did specifically consider developmental trauma is Mukaddes et al. (2004) which evaluated a 14-session psychoeducational program aimed at improving reciprocal-dyadic interaction between children and their caregivers for ten autistic boys (aged 2-5.5. years) and separately for 11 children (aged 2.5–5.8 years) with diagnoses of reactive attachment disorder (RAD). This study demonstrated greater changes in the RAD group compared to the autism group. However, none of the aforementioned studies or reviews related to children with the intersectional experiences of autism, developmental trauma, and attachment disruption.

Theraplay^®^ which is a physical, relationship-focused, playful and engaging treatment approach (Booth & Jernberg, 2010, p. xxi), that “…uses attachment-based play to create better relationships between parents and their children” (Booth & Koller, 1998: p. 308) is one such approach that has shown promise for the psychotherapeutic treatment of developmental trauma, attachment difficulties, and with neurodivergent populations. Theraplay^®^ aims to cultivate secure attachments, positive mental health, regulation, and positive internal working models by utilising play-based attachment-focused activities in the domains of structure (safety, organisation, and regulation), engagement (connection, optimum arousal, and shared joy), nurture (self-worth, empathy, and safe touch), and challenge (competence, confidence, and growth) see Booth and Jernberg (2010). Theraplay^®^ sessions are governed by the following nine core conditions: (1) direct, here-and-now experiences; (2) guided by the adults; (3) culturally sensitive; (4) preverbal, social, right-brained; (5) playful; (6) co-regulated; (7) responsive, attuned, empathic, and reflective; (8) interactive relationship-focused; (9) multi-sensory (Norris & Lender, 2020).

Colloquially, in the context of psychotherapy, “brain regions” refers to systems for emotional and sensory regulation, social engagement and stress responses shaped by early relationships. The “right brain” is seen to govern nonverbal communication, emotional attunement, body-based experience, and implicit regulation, which are fundamental for attachment but can be disrupted following traumatic experiences. The “left brain” is seen to support language and reasoning and is less accessible during periods of dysregulation. Schore (2022) proposes that early attachment experiences shape the development of right-hemisphere systems involved in affect regulation, nonverbal communication, bodily awareness, and social-emotional functioning. Interventions that emphasise nonverbal communication, emotional attunement, and embodied relational experiences may therefore support these functions. Theraplay^®^ is consistent with this theoretical perspective, as it relies on playful, attuned, and largely nonverbal interactions to promote feelings of safety, connection, and co-regulation and as such is often described as being a ‘preverbal, social, and right brained’ approach to psychotherapy. The language of “right-brained” and “left-brained” in this context is therefore analogous to emotional, instinctive processes and conscious processes respectively and is not meant to represent a clear distinction between the hemispheres.

Within the last decade, several prominent reviews have highlighted the promising nature of Theraplay^®^ in supporting parents and children with reducing the impact of developmental trauma and attachment disruptions. Chronologically, Stock et al. (2016) conducted a UK government-backed independent evidence review of post-adoption support interventions, reporting Theraplay^®^ to be a promising, research-based approach; Money et al. (2021) produced a systematic review examining Theraplay^®^ for children 12 years of age or below, reporting promising findings that suggest that Theraplay^®^ can reduce both externalising and internalising challenges in children; Purrington et al. (2023a) also reported preliminary positive support for use of Theraplay^®^ and play-therapy approaches when systematically reviewing family-based interventions for adoptive families; France et al. (2023) conducted a scoping review of Theraplay^®^ interventions, reporting that Theraplay^®^ is currently being utilised to support families with children presenting with a range of difficulties, predominantly resulting in positive post-therapy outcomes; Finally, Purrington et al. (2025a) completed an expert review presenting 41 Theraplay studies highlighting that adaptability of the Theraplay model for a diverse range of clinical presentations.

Additionally, Kalisch et al. (2023) published a scoping review of therapeutic supports for neurodiverse children who have experienced interpersonal trauma and found the literature favoured therapeutic approaches that relied less on verbal skills and that parent and caregiver involvement was repeatedly emphasised, each of these conditions align with the core principles within the Theraplay^®^ model.

Studies reviewing Theraplay^®^ outcomes and experiences report improved attachment relationships including relational closeness and trust (Purrington et al., 2023b; Salisbury, 2018; Salo et al., 2020; Smithee et al., 2021; Weir et al., 2020; Wettig et al., 2011), improved trauma symptomology (Eruyar and Vostanis 2020; Purrington et al. 2025a, b), improved communication and sociality (Francis et al., 2017; Siu, 2014), improved behavioural functioning (Francis et al., 2017; Rezaeianzadeh & Yazdanfar, 2024; Tucker et al., 2021; Weir et al., 2013), positive increases in regulation (Lindaman & Lender, 2009; Siu, 2023; Yazdanipour et al., 2022), and reduced internalised symptoms (Sepehrtaj et al., 2021; Siu, 2009).

Separately, Lindaman and Booth (2010) have presented a theoretical overview which provides a comprehensive rationale advocating for the use of Theraplay^®^ with autistic children and more recently Coleman and Hong (2023) presented a practitioner textbook detailing the use of integrative Theraplay^®^ for autistic children. However, empirical evidence for the application of Theraplay^®^ with autistic children remains relatively sparse, even more so for autistic children who have experienced developmental trauma and attachment disruptions.

Explorations of the use of Theraplay^®^ with autistic children and families include: Goodyear-Brown (2009) which presents two case studies within a practitioner textbook; Franklin et al. (2007) which explored eight children diagnosed with pervasive developmental disorder or mild-to-moderate autism and reports improvements in the parent-child relationship; Hiles-Howard et al. (2018) which examined eight autistic children engaging in two 1-hour Theraplay^®^ sessions a day for two weeks reporting improvements in child-parent interactions; Chang et al. (2021) which studied 20 autistic children engaging in 20 Theraplay^®^ sessions over a six month window, reporting improvements in social communication styles between children and parents; and Wettig et al. (2006) which tracked 56 children with ‘autistic like’ presentations who engaged in 26, 30-minute Theraplay^®^ sessions and reported reductions on clinical assessment scales. Additionally, Bundy-Myrow (2000) and Simeone-Russell (2011) have also presented clinical discussions for the use of Group Theraplay^®^ with autistic children. Collectively, these studies and clinical accounts suggest potential benefits of Theraplay^®^ for autistic children and their families; however, the literature is largely characterised by small samples, non-controlled designs, and practice-based reports, indicating a need for more rigorous and systematic research to establish effectiveness and generalisability.

There remains a lack of research on Theraplay^®^ for autistic children and their parents. Beyond this there is an absence of understanding regarding the efficacy of Theraplay^®^ for care experienced autistic children who have experienced developmental trauma and attachment disruptions. This is despite the presenting profiles of these experiences likely intersecting and contributing to increased difficulty for these parent-child dyads in developing attunement, intersubjectivity, and experiences of social co-regulation, all of which are seen as important facilitators for healthy attachment bonds. Moreover, a misconception persists in certain areas that Theraplay^®^ and Theraplay^®^-informed approaches may be unsuitable for autistic children with such concerns stemming from a perceived emphasis on touch and eye contact, which may be assumed to be dysregulating or uncomfortable for the cohort. It is therefore important to examine whether Theraplay can be effectively applied in a neurodiversity-affirming way to meet the needs of autistic children whilst achieving trauma- and attachment-related outcomes comparable to those seen in non-autistic cohorts. Therefore, the purpose of this retrospective service evaluation was to review the efficacy of a 30-hour neuro-collaborative Theraplay^®^-informed psychotherapeutic intervention on the presentations of developmental trauma and attachment difficulties in autistic looked after children who have experienced developmental trauma and attachment disruptions.

## Method

### Ethics

Ethical approval for this study was provided by the University of Salford, ethical approval reference 3171. Additionally, prior to the therapy, parents and caregivers were provided with consent forms and consented to the following statement:

- *At times*,* the data collected from questionnaires may be anonymised and used to evaluate the effectiveness of the work we offer*,* which may also contribute to published research. On these occasions*,* no personal information will be used*,* and only numerical data provided from the questionnaires will be included. This data will be collated with other anonymised data collected within the service.*

Additionally, all procedures contributing to this work complied with the American Psychological Association ethical standards in the treatment of participants, the British Psychological Society code of ethics and conduct, and the Health and Care Professionals Council standards of conduct performance and ethics.

### Service and Service Users

This service evaluation took place within Chrysalis Associates in the UK. Chrysalis Associates is a specialist developmental trauma and attachment service operating within four counties in the North of England for looked after and adopted children and adoptive families. The service is commissioned through local authority applications to the adoption support fund and referrals are made by each family’s social worker.

Service user data was eligible to be included within this service evaluation for all families who received a therapeutic package between March 2019 and March 2024 with children who had experienced developmental trauma and were showing signs of attachment difficulties as measured by the Marschak Interaction Method (MIM; Hitchcock et al., 2008). The MIM assesses the relationship quality between the caregiver and child, using play-based tasks to identify the caregivers’ capacity to provide structure, nurture, engagement and challenge. Adoptive parents also completed a pre-therapy clinical interview. Families were placed in the autistic group if their child had a diagnosis of autism as reported by their caregiver in the pre-therapy clinical interview (*N* = 35) and families with children without a diagnosis of autism were placed into the non-autistic group (*N* = 172).

The autistic group was 74.29% White British, 68.57% Male, and the average age at pre-therapy was 9.16 years, ranging from 4 to 17 years of age (*N* = 35, M = 9.16, SD = 3.37). Attendance rates within the autistic group ranged from 70% to 100% (*N* = 35, M = 96.60%). Within this group 23 families attended all 30 h of the therapeutic intervention (65.71%). The non-autistic group was 85.46% White British, 50.58% Male, and the average age at pre-therapy was 8.74 years, ranging from 3 to 16 years of age (*N* = 172, M = 8.74, SD = 3.06). Attendance rates within the non-autistic group ranged from 60% to 100% (*N* = 172, M = 96.16). Within this group 126 families attended all 30 h of the therapeutic package (71.59%). See Table 1 for sample demographic information.The legal status of the children varied including adoption (77.14% of the autistic group and 62.79% of the non-autistic group), special guardianship order (SGO; 11.42% of the autistic group and 17.44% of the non-autistic group) and care order (11.42% of the autistic group and 19.76% of the non-autistic group). Adoption permanently transfers parental responsibility to new parents, SGO provides a guardian parental responsibility while keeping birth family ties, and care order places the child in local authority care with shared parental responsibility.

**Table 1:** Sample demographic information

	Autistic Group (*N* = 35)	Non-Autistic Group (*N* = 172)	Difference
Age (Years)			
Mean	9.16	8.74	*t*=0.13, *p*=.90
Range	4–17	3–16	
Standard Deviation	3.37	3.06	
*Assigned Sex*			
Male	68.57%	50.58%	*χ2* = 3.38, *p*=.07
Female	31.43%	49.42%	
*Ethnicity*			
White British	91.40%	85.40%	*χ2* = 2.62, *p*=.62
Mixed race	5.70%	3.50%	
Asian	2.90%	1.80%	
Other white	0%	4.70%	
Unknown	0%	1.20%	
*Legal Status*			
Adopted	77.14%	62.79%	*χ2* = 1.36, *p*=.51
SGO	11.42%	17.44%	
Care Order	11.42%	19.76%	
*Attendance Rates*			
Mean	96.60%	96.16%	*t*=0.29, *p*=.77
Range	70%-100%	60%-100%	
Percentage of participants with 100% attendance	65.71%	71.59%	
Standard Deviation	7.55	8.00	

*SGO* special guardianship order

#### Neurocollaborative Therapeutic Intervention

The treatment approach is an integrative neuro-collaborative therapeutic method that consists of three components: (1) Family therapy session based on Theraplay^®^ concepts; (2) therapeutic parenting for the adoptive parents alone; and (3) sensory regulation sessions that include the parent and/or and the child. The treatment was designed in accordance with Perry’s (2009) neurosequential model of therapeutics which posits that psychotherapeutic interventions for the treatment of trauma in children should initially target the brain stem, followed sequentially by interventions that target the limbic system, followed sequentially by interventions that target the cortex. However, the neuro-collaborative model of therapeutic practice expands on this concept by adopting psychotherapy interventions which aim to address various brain regions of each child concurrently, rather than sequentially. The therapeutic package is distinguished from a neurosequential therapeutic model by being referred to as a ‘neuro-collaborative’ therapeutic approach. An earlier replication of this therapeutic framework has been subjected to evaluation, yielding positive outcomes, see Purrington et al. (2022).

The therapeutic package consisted of 10 h dedicated to Theraplay^®^-informed sessions which took place between the parent and child, 10 h dedicated to therapeutic parenting sessions for the parents only, and 10 h dedicated to a sensory regulation program which were either delivered to parents only, the parent-child dyad, or children only according to developmental age and stage of the child and presenting needs of the family unit.

This intervention was originally developed to encompass 30 sessions distributed across a 10-week timeframe, with a weekly commitment of three hours. However, following the COVID-19 pandemic, families developed increased agency relating to their therapeutic package due to the rise in online delivery formats. There is therefore an element of flexibility in how the 30 h of therapeutic work is accessed, however the Theraplay^®^-informed family therapy sessions must be completed alongside the therapeutic parenting sessions at a ratio of 1:1.

The delivery of these sessions was implemented through various modalities, including videoconferencing platforms, in person meetings, or a hybrid approach incorporating both methods. Most commonly, the Theraplay^®^-informed family therapy sessions took place in person and the therapeutic parenting sessions took place remotely.

At the start of each family’s therapeutic package, the therapist tailored the neuro-collaborative treatment program to align with the family’s personal goals. Generally, however, the neuro-collaborative intervention seeks to improve attachment bonds between parents and children, enhance regulation, and reduce levels of psychological suffering present in the family unit.

#### Theraplay^®^-Informed Sessions

The hour of family therapy included both the adoptive parent(s) and their child, alongside their trauma and attachment therapist. These sessions were grounded in principles of Theraplay^®^. Occasionally therapists integrated principles of sensory regulation, Dyadic Developmental Psychotherapy (DDP) or Eye Movement Desensitisation and Reprocessing (EMDR) by clinically assessing the therapeutic needs of the family. The family therapy sessions were Theraplay^®^-informed with activities taking place within the dimensions of engagement, nurture, structure, and challenge, tailored to the specific needs of both the parents and child (Booth & Jernberg, 2010), whilst also prioritising the nine core conditions of Theraplay^®^-informed practice: interactive and relationship based; direct here-and-now experiences; guided by the adult; responsive, attuned, empathic, and reflective; preverbal social right brain level of development; multisensory; playful; coregulated; and culturally sensitive.

#### Therapeutic Parenting

A total of 10 h of therapeutic parenting guided by the concepts of Theraplay^®^ parent-sessions and Dyadic Developmental Practice and Parenting were provided to parents. During these sessions, a comprehensive range of psychoeducational components were explored, centred around the PACE approach - Playfulness, Acceptance, Curiosity, and Empathy- as well as the concepts of therapeutic parenting. Each session included a review, assessing prior family therapy sessions often using video recordings, allowing space to enhance reflective capacity. This review also included deepening parental understanding through an in-depth exploration into their own attachment histories and how these directly intersect with their child’s behavioural presentation, alongside gaining strategies for parental emotional well-being.

#### Sensory Regulation

A total of 10 h of a sensory regulation therapy session was completed. These sessions were designed based on The Just Right State Program developed by Eadaoin Bhreathnach (see Hughes & Bhreathnach, 2018 online pdf pages 18–24) and the Alert Programme (Williams & Shellenberger, 1996; Gill et al., 2018). An Occupational Therapist designed the sensory regulation program, focusing on assisting families with establishing areas whereby they feel support is vital for their child’s regulation.

The intervention embodied several activities aimed at promoting co-regulation and self-regulation. These consisted of a ‘snack and chat’ approach using foods designed to assist emotional regulation, a body-based attachment program and story-book series, The Scared Gang, which educates children about self- regulation (Bhreathnach, 2008). Additionally, age-appropriate mindfulness exercises were conducted during the sessions. Families were educated and trained in these exercises to acknowledge their child’s sensory processing difficulties, thus enabling them to engage their child effectively while minimising the risk of sensory overload.

#### Therapist Characteristics

The trauma and attachment therapists who delivered the Theraplay-informed and Therapeutic Parenting components of the intervention were all trained to at least Theraplay level 1, 2, or practicum standard, at least DDP level 1, 2, or practicum standard, and those integrating EMDR were also accredited EMDR therapists. Clinical experience ranged from 0 to 5 years to 25–30 years working with developmental trauma and attachment difficulties. Registered professions included social work, occupational therapy, psychotherapy, and clinical psychology. A total 21 trauma and attachment therapists provided therapy during the data inclusion window. All trauma and attachment therapists were provided with regular supervision by an experienced clinical psychologist or a consultant clinical psychologist.

The therapists who delivered the sensory regulation component of the intervention were provided additional training in the sensory programs. Clinical experience ranged from 0 to 5 years to 5–10 years. Professional backgrounds included occupational therapists and graduate psychologists. A total 15 therapists provided the sensory regulation programs during the data inclusion window. Each of these therapists were provided with regular supervision by an experienced clinical psychologist or a consultant clinical psychologist. Formal treatment adherence and intervention fidelity checks did not take place. However, every therapist engaged in regular supervision with an experienced clinical psychologist or a consultant clinical psychologist. During these clinical supervision sessions each therapists therapeutic work was reflected on and reviewed in line with the therapeutic models being used.

### Data Collection

To evaluate the effectiveness of the therapeutic package, a between subject pretest- post-test design was implemented. Prior to the initiation of therapy, no more than 3 months in advance, families completed pre-assessment measures. Following this, they participated in the neurocollaborative therapeutic intervention. Post therapy measures were administered on the second to last Theraplay^®^-informed and therapeutic parenting session, with clients instructed to complete and return the measures in a two-week timeframe. The assessment measures included the Child Behaviour Checklist (CBCL), the Trauma Symptom Checklist for Young Children (TSCYC), the Behaviour Regulation Inventory for Executive Function 2 parent form (BRIEF-2), and the Assessment Checklist for Children (ACC).

### Measures

Across all outcome measures higher scores indicate greater levels of symptom severity. Therefore, reductions in scores indicate improvements have been made. For all assessment measures aged-normed T-Scores were utilised.

#### Trauma Symptom Checklist for Children (TSCYC)

The TSCYC is a 90-item measure designed for children aged 3 to 12 years. It employs a 4-point Likert scale to evaluate 9 clinical subscales: depression, anger, dissociation, sexual concerns, post-traumatic stress (PTS) intrusion, PTS-avoidance, PTS-arousal and PTS-total (Briere, 2005). Additionally, the TSCYC includes 2 internal validity scales that assess symptom over-reporting and atypical response patterns (e.g., under-reporting symptoms). The TSCYC has demonstrated a robust overall alpha reliability score of 0.87 (Briere et al., 2001) and exhibits convergent validity with the CBCL (Wherry et al., 2008).

The TSCYC scales of post-traumatic stress (total composite score), post-traumatic stress arousal, anxiety, and depression were selected for inclusion in this study based on the hypothesis that these scales cover core features that are increasingly recognised as prominent manifestations of developmental trauma in autistic children. Consequently, these scales were considered the most appropriate indicators of intervention-related change when comparing autistic and non-autistic looked after children. Moreover, the selected TSCYC subscales were chosen to assess internalising trauma-related symptoms, complementing the CBCL, which was used to capture externalising behavioural difficulties. This approach enabled distinct but theoretically related domains of functioning to be examined while minimising overlap between measures.

#### Behaviour Rating Inventory of Executive Function – 2nd Edition (BRIEF2)

The BRIEF2 parent form is a 63- item questionnaire designed for children aged 5 to 18 years. It utilises a 3-point Likert scale (Gioia et al., 2015) to evaluate executive functioning within 9 domains: Organisation of Materials, Plan/Organise, Task Monitor, Inhibit, Emotional Control, Self Monitor, Shift, Working Memory and Initiate. These elements are further divided in 3 primary components: emotions, cognitive and behavioural regulation. Additionally, the BRIEF2 incorporates 3 internal validity scales, assessing consistency in response, response patterns of a negative nature and infrequent responses (Hendrickson & McCrimmon, 2019). The BRIEF2 is acknowledged for its validity and reliability, demonstrating high interval consistency across its index scores, with scores ranging from 0.79 to 0.97 in both clinical and normative samples (Hendrickson & McCrimmon, 2019). Finally, it exhibits string test-retest reliability with correlation coefficients ranging from 0.67 to 0.79.

The BRIEF2 scale of Global Executive Composite (GEC) was selected for data inclusion and evaluation as it provides an overall index of executive functioning, reflecting the broad improvements in self-regulation anticipated following the intervention.

#### Child Behaviour Checklist (CBCL)

The CBCL is a 120- item questionnaire for children aged 3 to 5 years (young version) and 6 to 18 years. It applies a 3-point Likert scale (Achenback & Rescoria, 2001) to evaluate a child’s externalising, internalising and total problems. The CBCL also exhibits vigorous test-retest reliability, with correlational figures ranging between 0.93 and 1.00, as reported by Achenback & Rescoria (2001).

The CBCL scales of Total Problems (which is a total composite score), rule breaking behaviour, and oppositional defiant problems were selected for inclusion for this study because they assess global behavioural difficulties and externalising behaviours associated with emotional and relational dysregulation following developmental trauma. In autistic looked after children, trauma may amplify behavioural dysregulation beyond that attributable to autism alone, making these subscales particularly relevant indicators of intervention-related change when comparing to a non-autistic control group.

#### Assessment Checklist for Children (ACC)

The ACC is a 120-item psychiatric assessment completed for children aged between 5 and 10 years. The assessment measures behavioural traits, emotional states, and manners of relating to others (Tarren-Sweeney, 2007). Tarren-Sweeney (2007) reported the ACC’s total clinical score scale to have a high internal consistency (Cronbach’s alpha=0.96), and states that the ACC’s validity is supported due to high correlations between the ACC and CBCL total clinical scores (boys: *r*=.89; girls: *r*=.90).

The ACC scales of total clinical score (composite self-esteem scale), non-reciprocal, and indiscriminate were selected for inclusion in this study as each assesses attachment-related relational functioning and behaviours that are central to developmental trauma. These domains were considered the most relevant indicators of intervention-related change in autistic and non-autistic looked after children, as they reflect relational security and caregiver engagement rather than core autistic characteristics.

#### Administration of Assessment Measures

The four assessment measures each had differing age-ranges for valid inclusion: TSCYC: 3–12 years, ACC: 5–10 years, BRIEF: 5–18 years, CBCL: 6–18 years. Routine practice in the service was to administer assessment measures if they maintained clinical validity which resulted in families completing different combinations of outcome measures according to their child’s age. For example, children aged 3–4 years completed just the TSCYC, children aged 5 years completed the TSCYC and the BRIEF-2, children aged 6–10 years completed all four assessment measures, children aged 11–12 years completed the TSCYC, BRIEF, and CBCL, and children aged 13 + completed the BRIEF and CBCL. Most children included in this service evaluation were aged between 3 and 12 years and therefore the TSCYC was the most completed assessment measure followed by the BRIEF2, CBCL, and then the ACC.

It is also noted that a number of post-therapy questionnaires were returned with missing data which resulted in differing sample sizes between main scores and subscale scores being found in the ACC and TSCYC. In the autistic cohort one family returned the TSCYC with missing data in certain question items and one family returned the ACC with missing data in certain question items. In the non-autistic group six families returned the ACC with missing data in certain question items.

### Data Analysis

Two forms of data analysis were conducted in this study. First, in order to assess the overall effect of the treatment package, paired sample t-tests (1-tailed) with Cohen’s *d* as the measure of effect size were conducted to assess whether there was any change between pre-test scores and post-test scores following treatment for each outcome, for the full cohort and separately for the autistic and non-autistic groups. Second, Analysis of Covariance (ANCOVA) was conducted to assess whether there were any differences in change from pre-test to post-test between the two groups (autistic and non-autistic). Post-test score was included as the dependent variable and group (autistic or non-autistic) as the fixed factor. Pre-test score, gender, and age at beginning of treatment were included as covariates. Gender was included as a binary variable with female as zero (all participants identified as either male or female), and missing data were dealt with using listwise deletion. All data analyses were conducted using SPSS Version 29.0.

## Results

### Change from Pre-Test to Post-Test for Full Cohort and Separate Groups

For all outcome measures, the mean post-test score was lower than the mean pre-test score, indicating movement in the direction of improvement. Using an alpha level of 0.05, every outcome measure showed significant improvement for the full cohort. After adjusting the alpha level to 0.004 to correct for multiple tests (Bonferroni correction), total post-traumatic stress, post-traumatic stress arousal, CBCL total problems, and oppositional defiant problems were still significantly reduced. Effect sizes ranged between 0.17 and 0.34 indicating small effect sizes sometimes approaching medium (see Table 2). When separated in the autistic and non-autistic samples, these improvements tended to remain significant for the larger non-autistic group. No significant improvements were shown for the smaller autistic group, but the direction and effect sizes suggest that there may be some improvement that was not detected here due to the small sample size (see Table 3).

**Table 2:** Paired t-test results (full sample)

	*n*	Pre-test M (SD)	Post-test M (SD)	*p*	d
TSCYC					
Total Post-Traumatic Stress	171	67.37 (17.07)	64.24 (14.92)	< 0.001*	0.25
Post-Traumatic Stress Arousal	172	69.81 (17.66)	65.89 (17.74)	< 0.001*	0.30
Anxiety	172	59.37 (15.45)	57.31 (14.37)	0.01	0.17
Depression	171	60.61 (13.38)	58.56 (12.26)	0.01	0.18
CBCL					
Total Problems	137	73.50 (11.99)	70.77 (13.92)	< 0.001*	0.31
Rule Breaking Behaviour	137	68.89 (11.96)	67.13 (12.76)	0.004	0.23
Oppositional Defiant Problems	137	71.12 (11.86)	68.34 (12.74)	< 0.001*	0.34
BRIEF-2					
Global Executive Composite	164	70.80 (10.44)	69.23 (11.60)	0.008	0.19
ACC					
Total Clinical Score	118	62.28 (17.84)	59.57 (18.99)	0.01	0.22
Non-Reciprocal	125	59.73 (17.92)	56.78 (18.77)	0.02	19
Indiscriminate	124	54.33 (16.79)	51.66 (16.39)	0.006	0.23

* Significant after Bonferroni correction; *ACC* Assessment Checklist for Children, *BRIEF-2* Behaviour Rating Inventory of Executive Function 2nd Edition, *CBCL* Child Behaviour Checklist for Children, *TSCYC* Trauma Symptom Checklist for Young Children

**Table 3:** Paired t-test results (by group)

**Autistic group**	***n***	**Pre-test M (SD)**	**Post-test M (SD)**	***p***	**d**
TSCYC					
Total Post-Traumatic Stress	26	66.46 (13.88)	64.42 (11.98)	0.32	0.20
Post-Traumatic Stress Arousal	27	71.59 (16.62)	67.04 (13.58)	0.15	0.29
Anxiety	27	60.33 (14.04)	57.81 (13.53)	0.24	0.23
Depression	26	58.69 (12.34)	56.42 (10.10)	0.27	0.22
CBCL					
Total Problems	17	70.76 (9.18)	70.88 (10.66)	0.96	0.01
Rule Breaking Behaviour	17	67.53 (9.74)	64.65 (9.67)	0.05	0.53
Oppositional Defiant Problems	17	67.00 (10.22)	65.53 (10.62)	0.51	0.16
BRIEF-2					
Global Executive Composite	20	74.10 (6.66)	71.75 (9.87)	0.15	0.34
ACC					
Total Clinical Score	15	55.40 (15.79)	57.87 (12.61)	0.27	0.30
Non-Reciprocal	16	59.75 (15.43)	59.13 (16.88)	0.73	0.09
Indiscriminate	16	50.50 (17.65)	53.13 (16.55)	0.12	0.42
**Non-autistic group**	***n***	**Pre-test M (SD)**	**Post-test M (SD)**	***p***	***d***
TSCYC					
Total Post-Traumatic Stress	145	67.53 (17.61)	64.21 (15.42)	0.003	0.25
Post-Traumatic Stress Arousal	145	69.48 (17.89)	65.68 (18.44)	< 0.001*	0.30
Anxiety	145	59.19 (15.74)	57.22 (14.56)	0.06	0.16
Depression	145	60.96 (13.57)	58.94 (12.60)	0.04	0.17
CBCL					
Total Problems	120	73.88 (12.32)	70.75 (14.36)	< 0.001*	0.36
Rule Breaking Behaviour	120	69.08 (12.27)	67.48 (13.13)	0.029	0.20
Oppositional Defiant Problems	120	71.71 (11.99)	68.73 (13.00)	< 0.001*	0.37
BRIEF-2					
Global Executive Composite	144	70.34 (10.80)	68.88 (11.81)	0.04	0.17
ACC					
Total Clinical Score	103	63.28 (17.97)	59.82 (19.78)	0.008	0.27
Non-Reciprocal	109	59.72 (18.32)	56.43 (19.07)	0.04	0.20
Indiscriminate	108	54.90 (16.67)	51.44 (16.42)	0.003	0.29

* Significant after Bonferroni correction (*p*<.002); *ACC* Assessment Checklist for Children; *BRIEF-2* Behaviour Rating Inventory of Executive Function 2nd Edition, *CBCL* Child Behaviour Checklist for Children, *TSCYC* Trauma Symptom Checklist for Young Children

### Difference Between Groups

The analyses of covariance showed that group membership had no significant effect on post-test scores when controlling for pre-test scores (see Table 4).

**Table 4:** Analyses of covariance results

	df	F	*p*
TSCYC			
Total Post-Traumatic Stress	1, 158	0.06	0.81
Post-Traumatic Stress Arousal	1, 159	0.11	0.74
Anxiety	1, 159	0.002	0.97
Depression	1, 158	0.04	0.84
CBCL			
Total Problems	1, 130	1.77	0.19
Rule Breaking Behaviour	1, 130	0.69	0.41
Oppositional Defiant Problems	1, 130	0.11	0.74
BRIEF-2			
Global Executive Composite	1, 157	0.05	0.82
ACC			
Total Clinical Score	1, 109	1.18	0.28
Non-Reciprocal	1, 116	0.68	0.41
Indiscriminate	1, 115	2.34	0.13

*ACC* Assessment Checklist for Children, *BRIEF-2* Behaviour Rating Inventory of Executive Function 2nd Edition, *CBCL* Child Behaviour Checklist for Children, *TSCYC* Trauma Symptom Checklist for Young Children

## Discussion

The aim of this retrospective service evaluation was to review the efficacy of a 30-hour neuro-collaborative Theraplay^®^-informed psychotherapeutic intervention on the presentations of developmental trauma and attachment difficulties in autistic looked after children who have experienced developmental trauma and attachment disruptions.

Outcomes across measures of trauma-related stress and arousal, and behavioural difficulties showed reductions between the pre- and post- therapy measures for the full cohort, with the ANCOVA results demonstrating no significant difference between groups. When evaluated as separate groups based on the presence of an autism diagnosis, the (larger) non-autistic group also showed statistically significant improvements across trauma -related stress and behavioural difficulties, whilst the (smaller) autistic group did not show any statistically significant improvements. However, the effect sizes associated with these non-significant changes suggest that with a larger autistic group, these changes may reach statistical significance. Combined with the ANCOVA results showing no between-group differences in change from pre to post test, these somewhat ambiguous results do suggest that this therapeutic package may be effective for autistic individuals as well as non-autistic individuals. However, more work is needed to evaluate this conjecture.

Improvements post treatment were statistically significant in post-traumatic stress symptoms and behavioural problems for the full sample and for the non-autistic group. Whilst post-treatment executive function and outcomes measuring patterns of relating within primary attachment relationships also moved in the direction of improvement, they did not reach statistical significance in this study. This may be due to the setting of a high statistical bar due to multiple tests and studies with larger sample sizes should be conducted. Overall, these findings indicate that the 30-hour neuro-collaborative Theraplay^®^-informed intervention is effective for families with children who have experiences of developmental trauma and attachment disruption, and maybe for children who are also autistic.

### Trauma Symptoms

The TSCYC showed a significant reduction in Total Post Traumatic Stress and Arousal scores following therapy for the non-autistic group. However, both groups reported comparable small effect size improvements. The cognitive model of post-traumatic stress disorder (PTSD) suggests that traumatic stress is sustained by the presence of traumatic memories, negative cognitive appraisals about the event, the self, and the world around them, and subsequent counter-intuitive safety behaviours which become maintaining factors (Ehlers & Clark, 2000). For looked after children who have experienced developmental trauma and attachment disruptions, it is likely that their negative appraisals and maintenance behaviours, will be equally informed by their attachment experiences within primary caregiver relationships. The theory of attachment places the child-caregiver relationship as the cornerstone of an adaptive internal working model of self, other, and world (Ainsworth et al., 1978; Bowlby, 1969, 1973, 1980, 1991; Main, 1995, 1999). Therefore, children with attachment disruptions and attachment difficulties may present with more complex negative traumatic appraisals and maintenance behaviours.

Autistic looked after children with these experiences may face further complications as there is thought to be an overlap of behaviour caused by a response to trauma and caused by underlying autistic differences (Leedham et al., 2023). For clinicians it can therefore be difficult to engage in treatment planning for autistic children who have also experienced developmental trauma and attachment disruptions, as it is challenging to ascertain which aspects of their presentations can be explained through a trauma and attachment lens and which aspects can be explained through a neurodivergence lens (Mansour et al., 2025).

It is important to consider how both neurodevelopmental characteristics and environmental experiences shape the neural and physiological systems that support social engagement and attachment. Polyvagal Theory (Porges, 2011) suggests the social engagement system is underpinned by vagal pathways originating in the ventral vagal complex, which regulate autonomic states associated with calmness, reciprocity, and caregiver-child coregulation. In autistic children, differences in sensory processing, autonomic regulation, and social communication may influence functioning of this system, shaping how autistic children initiate, interpret, and respond to caregivers’ social cues. Similarly, early relational trauma may disrupt the development of caregiver-mediated regulation of arousal, limiting consistent engagement of these autonomic regulatory systems during social interaction. Importantly, this framework does not imply a single causal pathway to attachment differences but rather highlights how variations in autonomic regulation arising from different developmental origins may result in similar attachment patterns. Rutgers et al. (2004) reported lower rates of attachment security among children with greater cognitive impairment and more pronounced autism-related characteristics, suggesting that differences in autonomic and social engagement regulation may influence the expression and assessment of attachment rather than attachment capacity itself.

The findings of this study were ambiguous in relation to differences between autistic and non-autistic children in the reduction of trauma symptoms in response to the intervention. The flexibility of the Theraplay^®^-informed therapeutic aspect within this intervention is an important consideration that may be suitable for this population. Theraplay^®^-informed practice can be flexibly adapted to meet the child’s developmental age, stage, and current presentations in the room (Booth & Jernberg, 2010; Norris & Lender, 2020). Theraplay^®^ is also naturally strengths-based and in this way Theraplay^®^ can be seen to be a neurodiversity-affirming therapeutic approach. It is hypothesised that future studies will demonstrate improvements in autistic children due to this flexibility.

Theraplay^®^-informed practice can be seen to naturally intervene at the level of negative felt-sense appraisal in the cognitive conceptualisation of PTSD by focusing on the development of a safe environment, by helping carers to create structure around their child, and engage in ways in which the child can begin to build trust and feel safer in their environment (mitigating negative appraisals about others). Additionally, trust and safety are developed with structuring and nurturing experiences as the child is provided with embodied experiences of being cared for in a way which communicates that the child is worthy of the time and attention it takes to provide for their needs (mitigating negative appraisals about the self). Equally, Theraplay^®^-informed practice also intervenes with PTSD maintenance behaviours for example through engagement activities, the therapist helps the carer and child to stay connected and attuned for longer periods of time in this restorative environment (mitigating unhelpful maintenance behaviours). In the final pillar of Theraplay^®^, the child is given the opportunity to face manageable challenges with the help of others to further build up self-esteem and closeness. This very brief example conceptualises how Theraplay^®^-informed activities may reduce trauma symptoms for autistic and non-autistic children with developmental trauma and attachment experiences by flexibly mitigating negative appraisals and unhelpful maintenance behaviours. However, this is a purely theoretical speculation and further research is required in order to better ascertain the mechanisms underpinning the positive changes in both groups.

### Behavioural Presentations

The CBCL showed significant reductions on the total problems and oppositional defiance scale suggesting improved behavioural and emotional presentations in the non-autistic group. Whilst the changes did not reach statistical significance for the smaller autistic group, there was a moderate effect size in rule-breaking behaviour here. The neurodiversity movement promotes the view that neurological differences are a natural and valuable part of human variation rather than being deficits or disorders (Jaarsma & Welin, 2012; Kapp, 2020). This movement promotes acceptance of differences in social interaction, communication, thinking, and behaviour, rather than trying to change these differences to fit in with the norm. In addition, the social model of disability holds the perspective that individuals are disabled by their environment, and a lack of understanding and accommodations impacts on their ability to function (Dundon, 2023). Moreover, it recognises the role of society in supporting the needs of an individual and making environmental adaptations, including one’s own communication style, to reduce their challenges.

The neurocollaborative therapeutic intervention aimed to help parents better understand their child and develop connections in an alternative way via the use of Theraplay^®^-informed sessions, therapeutic parenting support, and a sensory intervention programme. It is assumed that the relatively large effect size for CBCL rule breaking behaviour in the autistic group does indeed represent an improvement, this could be related to parents having developed a greater understanding of their child’s presentation, preferences, and sensitivities, thus making it more likely for the parent to make the necessary adaptations to support their child’s health and wellbeing. Additionally, parents may have been better able to identify the imposed social and environmental barriers surrounding their child and remove them, further resulting in improved behavioural and emotional difficulties.

### Regulation & Executive Function

The reduction in Global Executive Composite (GEC) scores of the BRIEF2 did not reach statistical significance although there was movement in the direction of improvement for both separate groups, and no difference between the autistic and non-autistic groups. Interestingly, descriptively the GEC score for the autistic cohort improved at a greater rate compared to the non-autistic cohort and was seen to have a small-moderate effect. Larger studies are now required in order to appropriately explore this. However, Green’s (2023) model of “emergent” and “transactional” autistic experiences may be able to provide a theoretical conceptualisation of the small but positive improvements seen in this outcome measure. This model views autism as a natural transaction that occurs between neurodiverse brain differences, personal experiences of the world, and the emergent adaptive behavioural phenotype. The model acknowledges that autistic individuals experience an “autistic sensorium” of intense or dulled sensory experiences (their emergent autism experience), however as a result often face misunderstanding and stigma from others which can lead to interpersonal difficulties and the manifestation of transactional autistic experiences and symptoms (Green, 2023).

Thus, overwhelming environments and sensory overload from eye contact (emergent autism) can cause difficulty for autistic individuals in engaging with others, which can be negatively interpreted by non-autistic others, leading to transactional autism experiences as barriers to relationships developing (Clément et al., 2022). This model supports the concept that in the formative years when the developing neurodivergent brain interacts with distressing sensory and interpersonal environments, their emergent behavioural responses are then responded to by others in ways that either support and reduce their felt sense of difference or hinder and compound differences.

In line with this model, the neuro-collaborative therapeutic package utilises principles of sensory regulation targeting the brain stem and diencephalon alongside attachment-based play targeting the limbic system. Through this neuro-collaborative therapeutic approach it is hoped that the sessions can each provide an optimal sensory environment for the carer and child to allow the child to flourish in their natural differences and reduce the negative effects of transactional autism. More specifically, it can be hypothesised that the integration of sensory regulation and Theraplay^®^-informed practice supported the autistic children by utilising structure to emphasise predictability and clear boundaries, engagement to increase the time spent in regulated interaction, and nurture to facilitate a safe relationship between the parent and child. Franklin et al. (2007) found Theraplay^®^ to have a normalising effect on epinephrine levels and physiological stress of dysregulation in autistic children and their parents alongside improved parental affective expression and dyadic eye coordination suggesting improved coregulation. In other words, the parent’s improved regulation and experiences of their child increases their responsiveness to and understanding of their child who then in turn improves in their responsiveness to their parent.

The parents’ understanding of their children’s transactional autism and reduced physiological arousal allows for co-regulation. As the child learns to trust the parent and therapist to help them regulate, they begin to internalise this and regulate themselves, mirroring typical regulatory development. Emergent autism experiences are accommodated to facilitate regulation in an optimal environment, while transactional autism experiences, now better understood by the parent, are more appropriately environmentally managed.

Separately, the monotropic theory of autism suggests that autistic individuals, in comparison to non-autistic individuals, are more likely to demonstrate a narrow, intensely focused tunnel of attention (Murray et al., 2005). Therefore, cognitive resources are pooled into one or a few interests, resulting in behavioural presentations described in the diagnostic criteria: restricted, repetitive behaviours and interests, social and lingual communication differences, and hyper- and hypo- sensitivities. Experiences of developmental trauma and attachment disruption can inform this channel of attention. Subsequent to adverse attachment and trauma experiences, a child’s nervous system develops a hypervigilance on safety and survival, therefore narrow attention on repeated behaviours can also provide a soothing function or form a survival mechanism. Attempts to shift attention are difficult and distressing as they reduce the child’s control and their feeling of safety. Hypervigilance (trauma) and hypersensitivity (autism) exaggerate attention upon stimuli within the attentional channel producing overstimulation and overreaction. Stimuli outside the attentional channel is not registered and the child seems zoned out. This impacts social interaction where there is excessive incoming stimulation. Information such as eye contact can cause hyper-arousal while other information is lost, making social contact difficult and increasing misunderstanding. This reduces the quality of relationships and furthers the inability of the child to trust or understand others.

The Theraplay^®^-informed aspect of this intervention sought to support the parents to engage in regulating ‘moments of meeting’ with their child by synchronising into (and ‘rolling with’) their child’s channel of attention. Through these repeated experiences the carer becomes better able to understand their child’s monotropism, sensory experiences, trauma, and interests so that the child feels seen, heard, and understood, creating an opening for deepening attachment.

Previous research corroborates these mechanisms for change. Hiles Howard et al. (2018) found that Theraplay^®^ improves joint attention between autistic children and their parents. The 2-week intensive Theraplay^®^ course resulted in the parent learning to synchronise with the child’s behaviour according to their focus of attention which improved the child’s proximity to the parent and the child’s responsiveness. It was perceived that this improved joint attention and reduced parental stress relating to difficulties in attuning to their child’s behaviour (Hiles Howard et al., 2018).

Improved parental understanding may have also been enhanced due to the therapeutic parenting aspect of the neuro-collaborative intervention. In studies exploring parental lived experiences of dyadic developmental parenting sessions, parents reported that DDP helped them to develop an increased level of understanding of their child’s mind and behaviour (Agbayani, 2014; Turner, 2012; Wingfield & Gurney-Smith, 2019). Additionally, applying therapeutic parenting in a consultation format similar to the therapeutic parenting sessions utilised within this study has been found to provide reassurance and support whilst also cultivating enhanced caregiver understanding about the child in focus (Gosling & Purrington, 2023). This further evidences the value of working separately with parents and the parent-child dyad whilst completing therapeutic support in the area of neurodivergence, developmental trauma, and attachment disruption.

However, as mentioned above when considering the changes in regulation and executive function, although improvements were seen in the dataset these were not statistically significant which may be due to the small sample size. Larger studies are now required to fully investigate the mechanisms for change speculated above.

### Attachment Presentations

Scores on the ACC scales of Total Score, Indiscriminate, and Non-Reciprocal reduced at a statistically significant level for the non-autistic group with small effect sizes, but not for the autistic group,, additionally there was no statistical difference between the autistic and non-autistic groups. However, descriptively, the attachment presentations do present the only difference between the two groups within the study. The non-autistic group reported reductions in all three scales whereas the autistic group reported increases on the scales of total clinical score and indiscriminate and no real change on the non-reciprocal scale.

The ACC non-reciprocal scale relates to a child’s quality of interpersonal skills and behaviours such as appearing manipulative, uncaring, seeming to lack empathy or not showing affection whilst the indiscriminate scale related to behavioural presentations such as changing friends quickly or acting too friendly with strangers. The average post-therapy scores within the non-autistic group improved considerably compared to pre-therapy evaluations on these scales, whereas the average scores within the autistic group remained stable (non-reciprocal) or increased in severity (indiscriminate) at post-therapy.

The discrepancies in post-therapy score changes between the two groups on these specific scales is to be expected and corroborates that the study’s inclusion and exclusion criteria for each group was robust. Numerous studies highlight the positive impact of Theraplay^®^ on reducing non-reciprocal and indiscriminate behaviours for non-autistic children (Francis et al., 2017; Purrington et al., 2022; Siu, 2014; Weir et al., 2020; Wettig et al., 2011). Attachment theorists may conceptualise that children typically display behavioural presentations of appearing uncaring towards others or being over familiar with strangers due to having developed a negative internal working model resulting from difficult attachment and/or trauma-related early life experiences. Therefore, an attachment rich therapeutic experience such as Theraplay^®^ can understandably reduce these presentations.

Whereas autistic children can present as not showing affection and being socially different towards strangers due to their neurobiological make-up. More specifically, ‘deficits in social-emotional reciprocity’ and ‘in developing, maintaining, and understanding relationships’ are two diagnostic hallmarks of autism (American Psychiatric Association, 2013). Therefore, the impact of an attachment-rich therapeutic experience is likely to be reduced on this element for the autistic cohort. Moreover, it may be ineffective or even harmful for a therapeutic approach to focus on modifying this innate social approach. Instead, a neurodiversity-affirming stance towards autistic individuals should be sought during therapeutic work (Dundon, 2023). Therefore a lack of improvement on these scales is not surprising because the study was not focused on working with neurological differences but with healing trauma. The natural differences seen in autism are permanent. Despite this Theraplay remains effective in reducing symptoms of developmental trauma.

It is important to note that the absence of change on the ACC scales within the autistic group does not necessarily mean that autistic children and their parents within this study did not feel closer, more attuned, and more intersubjectively connected following the intervention. It is possible that the ACC is not sensitive enough to ascertain these changes within neurodiverse cohorts, reflecting a need for the development of autistic-informed assessment tools.

### Implications for Research and Practice

In certain areas of clinical practice there remains a common misconception that Theraplay^®^ and Theraplay^®^-informed practice cannot be used with autistic children who have experienced developmental trauma and attachment disruptions due to a misunderstood focus on touch and eye contact that may be experienced as dysregulating or re-traumatising for this cohort. This study attempted to demonstrate that Theraplay^®^-informed practice can be appropriately adapted in a neurodiversity-affirming way to meet the neurodiverse presentations of autistic children whilst continuing to obtain positive therapeutic outcomes on measures of trauma and attachment that are comparable to non-autistic cohorts. Whilst the measures did not yield significant improvements in the autistic group, the effect sizes and improvements in the full cohort combined with the non-significant ANCOVA suggest that this may have been due to the small sample size and that larger studies in the future can be predicted to yield significant improvements. Importantly, there was no suggestion that the therapeutic package led to any form of deterioration in functioning. Theraplay^®^ as a therapeutic offer could therefore be considered for autistic children with experiences of trauma and attachment difficulties and providers are encouraged to evaluate and publish their anonymised data to improve collective understanding of the efficacy of this therapeutic approach.

To develop on this understanding, qualitative research with carers of autistic children and the voices of autistic children who have experienced developmental trauma and attachment disruptions and received Theraplay^®^-informed practice is necessary to ascertain the acceptability and lived experiences of this intervention. Alongside this, it would be beneficial to interview therapists working with families with autistic looked after children to explore the adaptations made to Theraplay^®^-informed practice, therapeutic parenting, and sensory regulation work that support these models in their efficacy with this cohort.

### Limitations

As with all practice-based evidence this study is not without limitation, and the authors acknowledge the limitations that detract from the reliability and validity of the findings. The study did not include a randomisation process, has a relatively small autistic subgroup sample, there is considerable intervention heterogeneity making it difficult to isolate active treatment mechanisms, and the outcome data relies heavily on caregiver report which may have been vulnerable to expectancy effects.

Additionally, although all trauma and attachment therapists were trained in the core therapeutic modalities and supervised by a Clinical Psychologist, formal fidelity checks did not routinely take place and therefore it is not possible to comment on the quality of the therapeutic practice which took place relating to adherence to therapeutic modalities. Additionally, although Theraplay^®^-informed practice was the core modality within the family therapy sessions, it is once again impossible to ascertain how much sensory regulation, DDP, or EMDR was brought into sessions or not at all. In line with this, as the intervention itself is neurocollaborative and therefore integrative by design, it is impossible to ascertain which features of the intervention contributed to therapeutic change compared to others.

Separately, information about treatment dropout was not collected and therefore it is impossible to ascertain the feasibility and acceptability of the approach. Alongside this, information about existing mental health diagnoses for each child was not collected therefore it is impossible to determine whether certain extraneous variables moderated the impact of therapy for each child and family. The individual item scores for each participant were also not stored in such a way that an internal consistency analysis could be calculated for these samples, and this was not conducted at the time of data collection. It is therefore possible that the measures used in this study were lacking in validity and future studies should take care to store item responses in a way that facilitates this kind of analysis.

Finally, the differences in sample size between the autistic group and non-autistic group presents a further limitation to the study. Moreover, it is important to consider that the psychometric assessment questionnaires utilised within the study had not been uniquely adapted to the symptomatology of autistic children. The study presents a retrospective service evaluation and in routine practice the service utilised the four general measures outlined in the study therefore bespoke measures more sensitive to autism were not available. The measures used were also not age appropriate for the entire dataset resulting in inconsistent completion rates across measures. Further research exploring the impact of Theraplay for autistic cohorts who have experienced developmental trauma and attachment disruption should seek to include autism-specific and autism-sensitive measures and ensure that these evaluative tools can be adapted for all participants in the study.

### Conclusions

This retrospective service evaluation aimed to review the efficacy of a 30-hour neuro-collaborative Theraplay^®^-informed psychotherapeutic intervention on the presentations of developmental trauma and attachment difficulties in autistic looked after children who have experienced developmental trauma and attachment disruptions compared to non-autistic counterparts. Findings indicated significant improvements across the full cohort on measures of post-traumatic stress symptoms and emotional and behavioural difficulties with no difference between groups. Although the smaller autistic group did not yield evidence of significant improvements, effect sizes suggest a possible improvement that was not captured in this study due to insufficient statistical power. Additionally, improvements seen on measures of executive functioning and attachment did not reach the level of significance. More research, including structured clinical trials and evaluation of clinical data, is now required to assess whether autistic children benefit from this therapeutic approach, to further understand the mechanisms underpinning these changes, and to understand the lived experiences of this intervention for therapists, carers of autistic children, and the autistic children themselves.

## Data Availability

We do not have ethical approval to release or share the raw data from this study due to participant anonymity.

## References

[CR1] Achenbach, T. M., & Rescorla, L. A. (2001). *Manual for the ASEBA school-age forms and profiles*. University of Vermont Research Center for Children, Youth, and Families.

[CR2] Agbayani, C. (2014). *Exploration of attachment-focused family therapy utility: Parenting sexually abused adopted children*. (Publication No. AAI3636757) [Doctorate Thesis, Capella University, Minnesota]. ProQuest.

[CR3] Ainsworth, M. D. S., Blehar, M. C., Waters, E., & Wall, S. (1978). *Patters of attachment: A psychological study of the strange situation*. Lawrence Erlbaum.

[CR4] American Psychiatric Association. (2013). *Diagnostic and statistical manual of mental disorders (5th ed)*. American Psychiatric Pub. 10.1176/appi.books.9780890425596

[CR93] Bhreathnach, É. (2008). *The Scared Gang*. Aldertree Press.

[CR5] Booth, P. B., & Jernberg, A. M. (2010). *Theraplay*^®^*: Helping parents and children build better relationships through attachment-based play*. Jossey-Bass.

[CR6] Bowlby, J. (1969). *Attachment and loss. Vol 1. Attachment*. Basic Books.

[CR7] Bowlby, J. (1973). *Attachment and loss: Vol 2. Separation*. Basic Books.

[CR8] Bowlby, J. (1980). *Attachment and loss: Vol 3. Loss, sadness, and depression*. Basic Books.

[CR11] Bowlby, J. (1991). Post-script. In C. M. Parkes, J. Stevenson-Hinde, & P. Marris (Eds.), *Attachment across the life cycle* (pp. 293–297). Routledge.

[CR12] Briere, J. (2005). *Trauma symptom checklist for young children (TSCYC)*. Psychological Assessment Resources.

[CR13] Briere, J., Johnson, K., Bissada, A., Damon, L., Crouch, J., Gil, E., & Ernst, V. (2001). The trauma symptom checklist for young children (TSCYC): reliability and association with abuse exposure in a multi-site study. *Child Abuse and Neglect*, *25*(8), 1001–1014. 10.1016/s0145-2134(01)00253-8 11601594

[CR14] Bundy-Myrow, S. (2000). Group Theraplay^®^ for children with autism and pervasive developmental disorder. In E. Munns (Ed.), *Theraplay*^®^*Innovations in Attachment- Enhancing Play Therapy*. Jason Aronson pp. 301–320.

[CR15] Chang, Y., Kim, B., & Youn, M. (2021). Changes in children with Autism Spectrum Disorder after Theraplay^®^ application. *Journal of the Korean Academy of Child and Adolescent Psychiatry*, *32*(3), 112–117. 10.5765/jkacap.210001 PMC8262971 34285636

[CR16] Clément, M. A., Lee, K., Park, M., Sinn, A., & Miyake, N. (2022). The need for sensory-friendly zones: Learning from youth on the autism spectrum, their families, and autistic mentors using a participatory approach. *Frontiers in Psychology*, *13*(883331). 10.3389/fpsyg.2022.883331 PMC9254619 35800952

[CR18] Coleman, R. A., & Hong, R. (2023). *Integrative Theraplay*^®^* approach for children on the Autism spectrum: Practical guidelines for professionals*. Jessica Kingsley.

[CR19] Cyr, C., Euser, E. M., Bakermans-Kranenburg, M. J., & Van Ijzendoorn, M. H. (2010). Attachment security and disorganization in maltreating and high-risk families: A series of meta-analyses. *Development and Psychopathology*, *22*(1), 87–108. 10.1017/s0954579409990289 20102649

[CR20] Dubowitz, H., Papas, M. A., Black, M. M., & Starr, R. H. (2002). Child neglect: Outcomes in high-risk urban preschoolers. *Pediatrics*, *109*(6), 1100–1107. 10.1542/peds.109.6.1100 12042549

[CR21] Dundon, R. (2023). *A therapist’s guide to neurodiversity affirming practice with children and young people*. Jessica Kingsley.

[CR22] Ehlers, A., & Clark, D. M. (2000). A cognitive model of posttraumatic stress disorder. *Behaviour Research and Therapy*, *38*(4), 319–345. 10.1016/s0005-7967(99)00123-0 10761279

[CR23] Eruyar, S., & Vostanis, P. (2020). Feasibility of group Theraplay^®^ with refugee children in Turkey. *Counselling and Psychotherapy*, *20*, 626–637. 10.1002/capr.12354

[CR24] Felitti, V. J., Anda, R. F., Nordenberg, D., Williamson, D. F., Spitz, A. M., Edwards, V., & Marks, J. S. (1998). Relationship of childhood abuse and household dysfunction to many of the leading causes of death in adults: The adverse childhood experiences (ACE) study. *American Journal of Preventive Medicine*, *14*(4), 245–258. 10.1016/s0749-3797(98)00017-8 9635069

[CR25] France, L., McIntosh, S., & Woods, K. (2023). Using Theraplay^®^ to support children and families: a scoping review. *Early Child Development and Care*, *193*(9–10), 1097–1111. 10.1080/03004430.2023.2227775

[CR26] Francis, Y. J., Bennion, K., & Humrich, S. (2017). Evaluating the outcomes of a school based Theraplay^®^ project for looked after children. *Educational Psychology in Practice*, *33*(3), 308–322. 10.1080/02667363.2017.1324405

[CR27] Franklin, J., Moore, E., Howard, A., Purvis, K., Cross, D., & Lindaman, S. (2007). *An Evaluation of Theraplay*^®^*using a sample of children diagnosed with Pervasive Developmental Disorder (PDD) or Mild to Moderate Autism*. (Poster presentation at the American Psychological Association Conference, 2007).

[CR92] Gill, K., Thompson-Hodgetts, S., & Rasmussen, C. (2018). A critical review of research on the Alert Program®. *Journal of Occupational Therapy, Schools, & Early Intervention, 11*(2), 212–228.

[CR28] Gioia, G. A., Isquith, P. K., Guy, S. C., & Kenworthy, L. (2015). *Behavior rating inventory of executive function, second edition (BRIEF*^®^2). Psychological Assessment Resources Inc.

[CR29] Goodyear-Brown, P. (2009). Theraplay^®^ Approaches for Children with Autism Spectrum Disorders. In E. Munns (Ed.), *Applications of family and group Theraplay*^®^ (pp. 69–80). Jason Aronson.

[CR31] Gosling, J., & Purrington, J. (2023). Experiences of foster carers remotely accessing the children in care (CIC) consultation model during the Covid-19 pandemic: A service evaluation. *Adoption & Fostering*, *47*(2), 174–191. 10.1177/03085759231178315(Original work published 2023).

[CR30] Gosling, J., Purrington, J., & Hartley, G. (2023). Exploring the Lived Experiences of Autistic Women: A Thematic Synthesis. *Rev J Autism Dev Disord*. 10.1007/s40489-023-00367-5

[CR32] Green, J. (2023). Debate: Neurodiversity, autism and healthcare. *Child and Adolescent Mental Health*, *28*(3), 438–442. 10.1111/camh.12663 37438108

[CR34] Hartley, G., Sirois, F., Purrington, J., & Rabey, Y. (2024). Adverse Childhood Experiences and Autism: A Meta-Analysis. *Trauma Violence & Abuse*, *25*(3), 2297–2315. 10.1177/15248380231213314 38041427

[CR35] Hendrickson, N. K., & McCrimmon, A. W. (2019). Test review: Behavior rating inventory of executive function^®^, second edition (BRIEF^®^2) by Gioia, G. A., Isquith, P. K., Guy, S. C., & Kenworthy, L. *Canadian Journal of School Psychology*, *34*(1), 73–78. 10.1177/0829573518797762

[CR36] Hiles Howard, A. R., Lindaman, S., Copeland, R., & Cross, D. R. (2018). Theraplay^®^ impact on parents and children with autism spectrum disorder: Improvements in affect, joint attention, and social cooperation. *International Journal of Play Therapy*, *27*(1), 56–68. 10.1037/pla0000056

[CR90] Hitchcock, D. L., Ammen, S., O'Connor, K., & Backman, T. L. (2008). Validating the Marschak Interaction Method Rating System with adolescent mother-child dyads. *International Journal of Play Therapy, 17*(1), 24.

[CR37] Jaarsma, P., & Welin, S. (2012). Autism as a natural human variation: reflections on the claims of the neurodiversity movement. *Health Care Analysis*, *20*, 20–30. 10.1007/s10728-011-0169-9 21311979

[CR38] Kalisch, L. A., Lawrence, K. A., Baud, J., Spencer-Smith, M., & Ure, A. (2023). Therapeutic Supports for Neurodiverse Children Who Have Experienced Interpersonal Trauma: a Scoping Review. *Rev J Autism Dev Disord*. 10.1007/s40489-023-00363-9

[CR39] Kapp, S. (2020). Introduction. In Kapp, S. (Ed.), *Autistic Community and the Neurodiversity Movement: Stories from the Frontline*, (pp.1–19). Palgrave Macmillan. 10.1007/978-981-13-8437-0

[CR40] Leedham, A., Wicks, L., Laxton-Kane, M., Hammond, S., Broom, L., Tunnicliffe, P., Jones, T., & Enright, L. (2023). Considerations of differential diagnosis in the assessment of Autism Spectrum Disorder in young people under the age of 18. *Clinical Psychology Forum*, *362*, 18–33. 10.53841/bpscpf.2023.1.362.18

[CR89] Lindaman, S., & Lender, D. (2009). Theraplay with adopted children. *Applications of family and group Theraplay*, 57–68.

[CR42] Lindaman, S., & Booth, P. B. (2010). Theraplay^®^ for children with autism spectrum disorders. In P. B. Booth and A. M. Jernberg (Eds.), *Theraplay*^®^*: Helping parents and children build better relationships through attachment-based play. Jossey-Bass* pp. 301–358.

[CR43] Lupindo, B. M., Maw, A., & Shabalala, N. (2023). Late diagnosis of autism: exploring experiences of males diagnosed with autism in adulthood. *Curr Psychol, 42*, 24181– 24197 (2023). 10.1007/s12144-022-03514-z PMC9361958 35967496

[CR44] Main, M. (1995). Recent studies in attachment: Overview with selected implications for clinical work. In S. Goldberg, R. Muir, & J. Kerr (Eds.), *Attachment theory; Social, developmental, and clinical perspectives* (pp. 407–472). Analytic.

[CR45] Main, M. (1999). Epilogue. Attachment theory: Eighteen points with suggestions for future studies. In J. Cassidy, & P. R. Shaver (Eds.), *Handbook of attachment: Theory, research, and clinical applications* (pp. 845–888). Guilford.

[CR46] Mansour, H., Kurana, S., Eshetu, A., Hoare, S., El Baou, C., Arnold, I., Halys, C., Stewart, G. R., Desai, R., John, A., Mandy, W., O’Nions, E., & Stott, J. (2025). Prevalence of Post-Traumatic Stress Disorder (PTSD) in autistic children or young people (CYP) and adults: A systematic review and meta-analysis. *Clinical psychology review*, *120*, 102621. 10.1016/j.cpr.2025.102621 40783299

[CR47] Milot, T., Ethier, L. S., St-Laurent, D., & Provost, M. A. (2010). The role of trauma symptoms in the development of behavioural problems in maltreated preschoolers. *Child Abuse & Neglect*, *34*(4), 225–234. 10.1016/j.chiabu.2009.07.006 20303174

[CR48] Moran, H. J. (2010). Clinical observations of the differences between children in the autism spectrum and those with attachment problems: The Coventry Grid. *Good AutismPractice*, *11*(2), 46–59.

[CR49] Murray, D., Lesser, M., & Lawson, W. (2005). Attention, monotropism and the diagnostic criteria for autism. *Autism*, *9*(2), 139–156. 10.1177/1362361305051398 15857859

[CR50] National Institute for Health and Care Excellence guideline NG26 (2015). *Children’s attachment. Attachment in children and young people who are adopted from care, in care or at high risk of going into care.* https://www.nice.org.uk/guidance/ng26/26741018

[CR51] Norris, V., & Lender, D. (2020). *Theraplay*^®^*: The practitioners guide*. Jessica Kingsley.

[CR53] Ostfeld-Etzion, S. (2024). Treating Trauma and PTSD in Children with Autism Spectrum Disorder (ASD): Unique Challenges Call for an Active Role in Psychotherapeutic Interventions— Case Report. *Journal of Infant Child and Adolescent Psychotherapy*, *23*(4), 338–349. 10.1080/15289168.2024.2411668

[CR54] Parsons, S., McCullen, A., Emery, T., & Kovshoff, H. (2018). Awareness within local authorities in England of autism spectrum diagnoses of looked-after children. *British Educational Research Journal*, *45*(1), 99–116. 10.1002/berj.3485

[CR55] Peterson, J. L., Earl, R. K., Fox, E. A., Ma, R., Haidar, G., Pepper, M., Berliner, L., Wallace, A. S., & Bernier, R. A. (2019). Trauma and Autism Spectrum Disorder: Review, Proposed Treatment Adaptations and Future Directions. *Journ Child Adol Trauma*, *12*, 529–547. 10.1007/s40653-019-00253-5 PMC6901292 31819782

[CR56] Porges, S. W. (2011). *The Polyvagal theory: Neurophysiological Foundations of emotions, attachment, communication, and self-regulation*. Norton.

[CR57] Purrington, J., Glover-Humphreys, E., Edwards, H., & Hudson, M. (2022). The impact of a brief neuro-collaborative play-based intervention on the presentations of developmental trauma and attachment difficulties in adopted children: A service evaluation. *International Journal of Play Therapy*, *31*(4), 237–247. 10.1037/pla0000182

[CR58] Purrington, J., Goodall, S., & Lynch, J. (2023a). Family-based psychological interventions for domestically adoptive families: a systematic review. *European Child And Adolescent Psychiatry*, *33*, 1239–1256. 10.1007/s00787-023-02210-y 37079133

[CR59] Purrington, J., Hartley, G., & Lyne, N. (2023b). Service users’ experiences of a 6-week remotely delivered Theraplay^®^ -informed group intervention for carers: A qualitative service evaluation. *International Journal of Play Therapy*, *32*(3), 181–195. 10.1037/pla0000201

[CR60] Purrington, J., Lindaman, S., & Jernberg, J. (2025a). Healing through play: Theraplay as an integrative therapy for parents and caregivers. *The American Journal of Medicine*, *9343*(25), 00828–00829. 10.1016/j.amjmed.2025.11.027 41354386

[CR61] Purrington, J., Price, A. D., Godfrey, C., Lynch, J., Cook, P. A., & Mukherjee, R. A. S. (2025b). The Impact of a Neurocollaborative Theraplay^®^ informed intervention on the presentations of developmental trauma and attachment difficulties in adopted children with prenatal alcohol exposure: An extended case study. *Journ Child Adol Trauma*, *2025*. 10.1007/s40653-025-00715-z PMC12433427 40955394

[CR62] Rezaeianzadeh, Z., & Yazdanfar, F. (2024). Effectiveness of Theraplay^®^ and filial therapy on aggression in preschool boys. *Current Psychology*, *43*, 17602–17613. 10.1007/s12144-024-05743-w

[CR63] Robertson, S. M. (2009). Neurodiversity, quality of life, and autistic adults: Shifting research and professional focuses onto real-life challenges. *Disability Studies Quarterly*, *30*(1). 10.18061/dsq.v30i1.1069

[CR64] Rutgers, A. H., Bakermans-Kranenburg, M. J., van Ijzenboorn, M. H., & van Berckelaer- Onnes, I. A. (2004). Autism and Attachment: A Meta-Analytic Review. *Journal of Child Psychology and Psychiatry*, *45*(6), 1123–1134. 10.1111/j.1469-7610.2004.t01-1-00305.x 15257669

[CR65] Salisbury, S. (2018). Using attachment enhancing activities based on the principles of Theraplay^®^ to improve adult-child relationships and reduce a child’s ‘overall stress’ as measured by the Strength and Difficulties Questionnaire (SDQ). *Emotional and Behavioural Difficulties*, *23*(4), 424–440. 10.1080/13632752.2018.1497000

[CR66] Salo, S., Flykt, M., Mäkelä, J., Lassenius-Panula, L., Korja, R., Lindaman, S., & Punamäki, R. L. (2020). The impact of Theraplay^®^ therapy on parent-child interaction and child psychiatric symptoms: a pilot study. *International Journal of Play*, *9*(3), 331–352. 10.1080/21594937.2020.1806500

[CR67] Sarr, R., Spain, D., Quinton, A. M. G., Happé, F., Brewin, C. R., Radcliffe, J., Jowett, S., Miles, S., González, R. A., Albert, I., Scholwin, A., Stirling, M., Markham, S., Strange, S., & Rumball, F. (2024). Differential diagnosis of autism, attachment disorders, complex post-traumatic stress disorder and emotionally unstable personality disorder: A Delphi study. *British Journal of Psychology*, *116*. 10.1111/bjop.12731 PMC11724683 39300915

[CR68] Schore, A. (2022). Right brain-to-right brain psychotherapy: recent scientific and clinical advances. *Annals of general psychiatry*, *21*(1), 46. 10.1186/s12991-022-00420-3 PMC9675148 36403062

[CR69] Sepehrtaj, A., Younesi, S. J., Seyed-Mousavi, P., Khani-Jeihooni, A., & Jafari, P. (2021). Effectiveness of Theraplay^®^ in Internalizing and Externalizing Problems in Bereaved Siblings of Children with Cancer. *Iran J Psychiatry Beha Sci*, *15*(2), e103992. 10.5812/ijpbs.103992

[CR70] Simeone-Russell, R. (2011). A practical approach to implementing Theraplay^®^ for children with autism spectrum disorder. *International Journal of Play Therapy*, *20*(4), 224–235. 10.1037/a0024823

[CR71] Siu, A. F. Y. (2009). Theraplay^®^ in the Chinese world: An intervention program for Hong Kong children with internalizing problems. *International Journal of Play Therapy*, *18*(1), 1–12. 10.1037/a0013979

[CR72] Siu, A. F. Y. (2014). Effectiveness of Group Theraplay^®^ on enhancing social skills among children with developmental disabilities. *International Journal of Play Therapy*, *23*(4), 187–203. 10.1037/a0038158

[CR73] Siu, A. F. Y. (2023). Evaluating two play-based approaches for children and carers in residential settings: A mixed-method, randomized control study. *International Journal of Play Therapy*, *32*(2), 63–74. 10.1037/pla0000130

[CR74] Smithee, L. C., Krizova, K., Guest, J. D., & Pease, J. C. (2021). Theraplay^®^ as a family treatment for mother anxiety and child anxiety. *International Journal of Play Therapy*, *30*(3), 206–218. 10.1037/pla0000153

[CR75] Stack, A., & Lucyshyn, J. (2019). Autism Spectrum Disorder and the Experience of Traumatic Events: Review of the Current Literature to Inform Modifications to a Treatment Model for Children with Autism. *Journal Of Autism And Developmental Disorders*, *49*, 1613–1625. 10.1007/s10803-018-3854-9 30539370

[CR76] Stock, L., Spielhofer, T., & Gieve, M. (2016). Independent evidence review of post-adoption support interventions. *Department for Education Research Report.* 5th July 2016. https://www.gov.uk/government/publications/post-adoption-support-interventions-independent-evidence-review

[CR77] Tarren-Sweeney, M. (2007). The assessment checklist for children — ACC: A behavioral rating scale for children in foster, kinship and residential care. *Children and Youth Services Review*, *29*(5), 672–691. 10.1016/j.childyouth.2007.01.008

[CR78] Tucker, C., Schieffer, K., Lenz, S., & Smith, S. (2021). Sunshine Circles: Randomized Controlled Trial of an Attachment-Based Play Group with Preschool Students Who are At-Risk. *Journal of Child and Adolescent Counselling*, *7*(3), 161–175. 10.1080/23727810.2021.1940658

[CR79] Turner, K. (2012). *Adoptive mothers’ experiences of dyadic developmental psychotherapy: Unpacking the invisible suitcase. (*Publication No. uk.bl.ethos.575385). [Doctorate Thesis, University of Nottingham]. EThOS: https://ethos.bl.uk/OrderDetails.do?uin=uk.bl.ethos.575385

[CR80] van der Kolk, B., & D’Andrea, A. (2010). Towards a developmental trauma disorder diagnosis for childhood interpersonal trauma. In R. A. Lanius, E. Vermetten, & C. Pain (Eds.), *The impact of early life trauma on health and disease: The hidden epidemic* (pp. 57–68). Cambridge University Press. 10.1017/CBO9780511777042.008

[CR81] Weir, K. N., Lee, S., Canosa, P., Rodrigues, N., McWilliams, M., & Parker, L. (2013). Whole Family Theraplay^®^: Integrating Family Systems Theory and Theraplay^®^ to Treat Adoptive Families. *Adoption Quarterly*, *16*(3–4), 175–200. 10.1080/10926755.2013.844216

[CR82] Weir, K., Pereyra, S., Crane, J., Greaves, M., Childs, T. S., & Weir, A. B. (2020). The Effectiveness of Theraplay^®^ as a Counselling Practice With Mothers and Their Children in a Substance Abuse Rehabilitation Residential Facility. *The Family Journal*, *29*(1), 115–123. 10.1177/1066480720980988

[CR84] Wettig, H. H. G., Franke, U., & Fjordbak, B. S. (2006). Evaluating the effectiveness of Theraplay^®^. In C. E. Schaefer, & H. Kaduson (Eds.), *Contemporary Play Therapy* (pp. 103–135). The Guilford Press.

[CR83] Wettig, H. H. G., Coleman, A. R., & Geider, F. J. (2011). Evaluating the effectiveness of Theraplay^®^ in treating shy, socially withdrawn children. *International Journal of Play Therapy*, *20*(1), 26–37. 10.1037/a0022666

[CR85] Wherry, J. N., Graves, L. E., & King, H. M. (2008). The convergent validity of the trauma symptom checklist for young children for a sample of sexually abused outpatients. *Journal of Child Sexual Abuse*, *17*(1), 38–50. 10.1080/10538710701884441 19842317

[CR91] Williams, M. S., & Shellenberger, S. (1996). *How does your engine run?: A leader's guide to the alert program for self-regulation*. TherapyWorks, Inc..

[CR86] Wingfield, M., & Gurney-Smith, B. (2019). Adoptive parents’ experiences of dyadic developmental psychotherapy. *Clinical Child Psychology and Psychiatry*, *24*(4), 661–670. 10.1177/135910451880773 30354281

[CR87] Yazdanipour, M., Ashori, M., & Abedi, A. (2022). Impact of group Theraplay^®^ on the social– emotional assets and resilience in children with hearing loss. *International Journal of Play Therapy*, *31*(2), 107–118. 10.1037/pla0000175

[CR88] Zainab Al-Attar, & Worthington, R. (2024). Trauma or autism? – understanding how the effects of trauma and disrupted attachment can be mistaken for autism. *Advances in Autism*, *10*(3). 10.1108/aia-07-2023-0041

